# Editorial: Immune plasticity and cancer: augmenting therapeutic potential of immunotherapy against tumor diseases

**DOI:** 10.3389/fimmu.2026.1922899

**Published:** 2026-07-14

**Authors:** Hardeep Singh Tuli, Hridayesh Prakash, Aklank Jain

**Affiliations:** 1 Department of Biosciences and Technology, Maharishi Markandeshwar (Deemed to be University), Ambala, India; 2 Centre of Excellence in Computational Research and Drug Discovery, Maharishi Markandeshwar (Deemed to be University), Ambala, Haryana, India; 3 Amity Centre for Translational Research (ACTR), Amity University Uttar Pradesh, Noida, Uttar Pradesh, India; 4 Non-coding RNA and Cancer Biology Lab, Department of Zoology, Central University of Punjab, Bathinda, Punjab, India

**Keywords:** cancer immunotherapy, immune evasion, immune plasticity, tumor heterogeneity, tumor microenvironment

Despite great breakthroughs in diagnosis, prevention and treatment, cancer is still one of the top causes of illness and mortality globally. In the last decade, immunotherapy has revolutionized the field of cancer management with durable clinical responses and long-term survival in patients with a variety of malignant diseases. However, the efficiency of contemporary immunotherapeutic methods is still limited by therapy resistance, tumor heterogeneity and immune evasion. In this environment, immunological flexibility has become a crucial predictor of cancer progression and therapy outcomes.

Immune plasticity is the striking ability of immune cells to change their phenotypic, function, and metabolic status in response to environmental signals. Within the tumor microenvironment (TME), immune cells are in continual interaction with cancer cells, stromal elements, and soluble mediators, producing complicated and often opposing immune responses. Immune plasticity can support efficient anticancer immunity, but can also increase immunosuppression, tumor growth, and resistance to therapy. Therefore, unravelling the mechanisms that dictate these dynamic shifts is critical for the development of next-generation cancer immunotherapies.

In order to provide a thorough forum for examining the complex relationship between immunological plasticity and cancer biology, the Research Topic, “Immune Plasticity and Cancer: Augmenting Therapeutic Potential of Immunotherapy against Tumor Diseases,” was created. This the Research Topic’s pieces highlight recent advances in our understanding of tumor-immune interactions, immune cell reprogramming, and therapeutic approaches created to use immunological plasticity for improved clinical benefit.

Several papers have addressed the contribution of important immune populations to the immunological landscape of malignancies. These studies demonstrate that the plasticity in phenotype and function within these cell types can either support or inhibit anticancer responses. For example, Yin et al. reviewed potential prognostic biomarkers in antitumor immunotherapy. Likewise, He et al., reviewed that targeting innate immune cells to generate anti-tumor immune responses holds great promise for improving immunotherapeutic approaches in cSCC. Liu et al., review suggested that SUMOylation inhibition as a promising adjunct to immunotherapy for tumor management. Tang and Yu highlighted the possible synergistic mechanism of the combined use of small molecule targeted medicines and CAR-T cell treatment. Similarly, Guo and Li examined that combination has shown good clinical performance with manageable safety profiles in cancer therapy.

The emerging research in this the Research Topic reveals that immune-cell destiny is not pre-determined but rather influenced by metabolic, epigenetic and cytokine-mediated signals generated within the TME. You et al. research article demonstrated cost-effectiveness analysis of benmelstobart, anlotinib and chemotherapy in extensive-stage small-cell lung cancer. As per the study, from the Chinese healthcare system’s perspective, BEN-AL-EC as a first-line treatment for ES-SCLC is unlikely to be cost-effective compared with PLB-EC. According to Chettri et al., CAR-Macrophage (CAR-M) therapy would be a potential asset of immunotherapy for numerous solid malignancies. Wang et al. have proposed ICGA-A and CRA as promising adjuncts in the treatment of TNBC. A further study by Deng et al. showed the dynamic biological alterations and dedifferentiation states of tumor subtypes during the progression process and offered useful recommendations for future therapeutic options.

We thank all the contributors for their excellent research and ideas offered to this the Research Topic as Guest Editors. We would also want to sincerely thank the reviewers for their knowledge, constructive assessments and commitment to ensuring scientific quality. Finally, we thank the editorial team and publishing staff for their continued support in preparing this the Research Topic.

We hope that the contributions in this the Research Topic will drive more study on the mechanisms of immunological plasticity and novel therapeutic options for cancer treatment. Understanding the dynamic interplay between tumors and the immune system will get us closer to realizing the full potential of immunotherapy in the battle against cancer.

